# Bilateral jaw dislocation following botulinim toxin type A treatment for chronic migraine

**DOI:** 10.1186/1129-2377-1-S14-P211

**Published:** 2013-02-21

**Authors:** RB Forbes, E Devenney

**Affiliations:** 1 Craigavon Area Hospital, UK

## Background

Botulinim Toxin Type A is a licenced treatment for chronic migraine. We describe a case of bilateral jaw dislocation which occurred following Botulinim Toxin Injection. Case Description The patient is a 46 year old lady with a 25 year history of chronic migraine and no previous history of joint dislocations. She was injected following the 155 unit fixed-dose, fixed site protocol with no immediate complications. 8 days later the patient developed acute bilateral jaw pain and an inability to close the mouth. An x-ray confirmed bilateral temporomandibular joint dislocations and this was reduced the following day by an oral surgeon. There has been no recurrence, however she continues to complain of jaw pain. As her initial injection was associated with an improvement in headache, she consented to further treatment on the basis that the temporalis muscle was not injected. To date there has been no recurrent dislocation.

## Conclusion

We believe that injection of the temporalis muscle in this patient predisposed to bilateral temporomandibular joint dislocation. To our knowledge this has not previously been reported. Regulatory authorities have been notified but treating neurologists need to be aware of this rare occurence.

